# Potential short-term effects of earthquake on the plant–soil interface in alpine grassland of the Qinghai–Tibetan Plateau

**DOI:** 10.3389/fpls.2023.1240719

**Published:** 2023-10-17

**Authors:** Hui Zuo, Hao Shen, Shikui Dong, Shengnan Wu, Fengcai He, Ran Zhang, Ziying Wang, Hang Shi, Xinghai Hao, Youquan Tan, Chunhui Ma, Shengmei Li, Yongqi Liu, Feng Zhang, Jiannan Xiao

**Affiliations:** ^1^ School of Grassland Science, Beijing Forestry University, Beijing, China; ^2^ Department of Natural Resources, Cornell University, Ithaca, NY, United States; ^3^ School of Environment, State Key Joint Laboratory of Environmental Simulation and Pollution Control, Beijing Normal University, Beijing, China

**Keywords:** earthquake, alpine grasslands, soil properties, plant nutrient, community diversity, productivity

## Abstract

Earthquakes are environmental disturbances affecting ecosystem functioning, health, and biodiversity, but their potential impacts on plant–soil interface are still poorly understood. In this study, grassland habitats in areas near and away from the seismo-fault in Madou, a region typical of alpine conditions on the Qinghai–Tibetan Plateau, were randomly selected. The impacts of earthquake on soil properties and plant nutrient content in the short term were emphasized, and their potential relationships with community diversity and productivity were examined. According to the findings of the study, the Maduo earthquake led to a decrease in soil nutrient content in alpine grassland ecosystems, especially soil TC, TN, TP, TCa, AP, AK, NH_4_^+^-N, and SOC, and inhibited the absorption of N, Ca, and Mg nutrients by plants. In addition, the diversity and productivity of communities were affected by both direct and indirect earthquake pathways. The negative impacts of seismic fracture on soil structure had the most significant direct impact on plant community diversity. Earthquakes also indirectly reduced community productivity by reducing the soil N content and inhibiting the absorption of plant nutrients. Our findings suggested that earthquakes could potentially decrease the stability of the alpine grassland ecosystem on the QTP by affecting nutrient availability at the plant–soil interface.

## 1 Introduction

Earthquake, a classic disastrous disturbance event, has a significant influence on global biodiversity, causing ecosystem structure and function to degrade and vegetation to be destroyed (Zhang et al., 2011; Cui et al., 2012; Su et al., 2015; Vaux et al., 2022). Disturbances of varying intensities and frequencies not only influence plant diversity and spatial distributions but also affect physiological and ecological processes in plants, and serve as forces of selectivity in their composition (Sousa, 1984; Lindenmayer et al., 2010; Loto and Bravo, 2020). To date, the response mechanism of vegetation after earthquake has remained a topical issue in ecology (Huang et al., 2017; Kang et al., 2021; Kang et al., 2022). Community diversity as well as species richness can be significantly reduced by earthquake, leading to a decline in productivity and serious damage to grassland ecosystems (Mittelbach et al., 2001; Ouyang et al., 2008; Zhang et al., 2011). Eleven years following the Wenchuan earthquake, herbaceous communities still dominated the land recovery, and plant diversity and species evenness were still significantly reduced (Kang et al., 2021). Plant functional traits at the community level that covered multiple organs of grassland plants, including seeds, leaves, stems, and roots, differed markedly compared to plants not affected by the earthquake (Kang et al., 2022). However, we have not gained a clear understanding of what causes the reduction in grassland community diversity and productivity after earthquakes.

Earthquakes may affect community aggregation by completely altering soil structure and nutrient composition, thus affecting community diversity and productivity (Kang et al., 2021). Changes in species diversity can reflect variations in community structure and composition, which are strongly driven by soil factors (Xu et al., 2019). The disturbance of soil structure and nutrients after earthquakes can affect vegetation structure and function, further leading to a reduction in community diversity and loss of vegetation biomass (Zhang et al., 2011; Cheng et al., 2012; Cui et al., 2012). During a major earthquake, original nutrient-rich and well-formed soils are susceptible to disturbance by seismic geological hazards, which can damage the surface soil structure and cause nutrient loss (Lin et al., 2017). In alpine grassland ecosystems, plant productivity is significantly related to soil nutrient status (Xu et al., 2019).

Variations in soil nutrient levels alter the nutrient uptake strategies of plants, while plant nutrient uptake, transport, and utilization control the development and production of plants (Güsewell and Koerselman, 2002; Yan et al., 2019). Despite the fact that plants with high chemical equilibrium can keep nutrient concentrations throughout their tissues largely constant to make adjustments to changes in the environment, reductions in available nutrients can still restrict the development of dominant species and thus impact growth and stabilization of plant communities (Yu et al., 2011; Yu et al., 2015). The plant traits relating to nutrient acquisition and utilization are strongly affected by soil fertility, which results in a close relationship with community diversity and productivity (Zemunik et al., 2015). The change in plant nutrient uptake is one of the mechanisms that explain the connection of biodiversity and primary production (Hooper et al., 2005). At this stage, plant nutrient uptake is being applied to solve ecological issues related to plant development and plant response to soil nutrient variations (Zhang et al., 2019).

The Qinghai–Tibetan Plateau (QTP), located at the boundary between lithospheric plates, is prone to be affected by strong earthquakes (Fattorini et al., 2018; Chen et al., 2021). Alpine grassland is the most important ecosystem to maintain the gene pool of biodiversity of the QTP, and it is usually affected by such disturbance (Dong et al., 2013; Dong et al., 2020). In a previous study, we discovered that the 7.4 Maduo earthquake in May 2021 caused a marked negative effect on alpine grassland plant communities, resulting in diversity loss and productivity decline (Zuo et al., 2022). However, the potential mechanism related to this phenomenon remains unclear. Soil structure and nutrient content will be altered after an earthquake, which will lead to an impact on the plant root system’s uptake of soil nutrients (Guo et al., 2013; Lin et al., 2017; Briat et al., 2020). Plant uptake of nutrients is closely linked to the diversity and productivity of the community (Fay et al., 2015; Lü et al., 2019). On this basis, we performed this research to investigate the potential short-term effects of earthquake on the plant–soil interface of alpine grassland with three hypotheses: (1) soil nutrient will be lost due to earthquake disturbance in alpine grassland; (2) the plant nutrient uptake capacity of alpine grassland plants will be limited under the short-term effect of earthquake rupture; and (3) the earthquake inhibits the plant uptake of nutrients by affecting soil nutrients, which is one of the major causes for the reduction in plant diversity and productivity in the long run. Our goal in this study is to inform the recovery and ecological construction of alpine grassland after earthquake and help us understand the adaptive strategies of alpine species responding to the changed environment.

## 2 Materials and methods

### 2.1 Field site

The field site of Maduo County of Qinghai Province, China (96°50′–99°20′ E, 33°50′–35°40′ N), is located in the Yellow River source area in the northeastern part of the QTP. It is an important ecological barrier on the QTP, with an approximate total area of 25,300 km^2^ and an altitude mostly between 4,200 and 4,800 m. Maduo County has an alpine grassland climate with an average annual temperature of −4.1°C, a potential annual evaporation of 1,264 mm, and an average annual precipitation of approximately 303.9 mm, 86% of which mainly happens between May and September (Zhang et al., 2010). The vegetation types are mainly grassland, including alpine steppes and alpine meadows, and the kobresia species of the sedge family such as *Kobresia humilis*, *Kobresia tibetica*, and *Kobresia pygmaea* are dominant.

Maduo County is situated within the historically seismically active Bayan Har Block in Qinghai, Province, China, which was struck by an M*w* 7.4 earthquake on 22 May 2021, at a depth of approximately 17 km. The Maduo earthquake was the biggest earthquake on the Chinese mainland following the Wenchuan earthquake in 2008. The earthquake fault spread bilaterally toward both the east and the west, with a rupture length of approximately 170 km and a duration of approximately 38 s, causing the formation of several new seismic fault zones that severely endangered the fragile alpine grassland ecosystem (Feng et al., 2022; Zhang et al., 2022; Zuo et al., 2022).

### 2.2 Field vegetation and soil sampling

**Figure 1:**
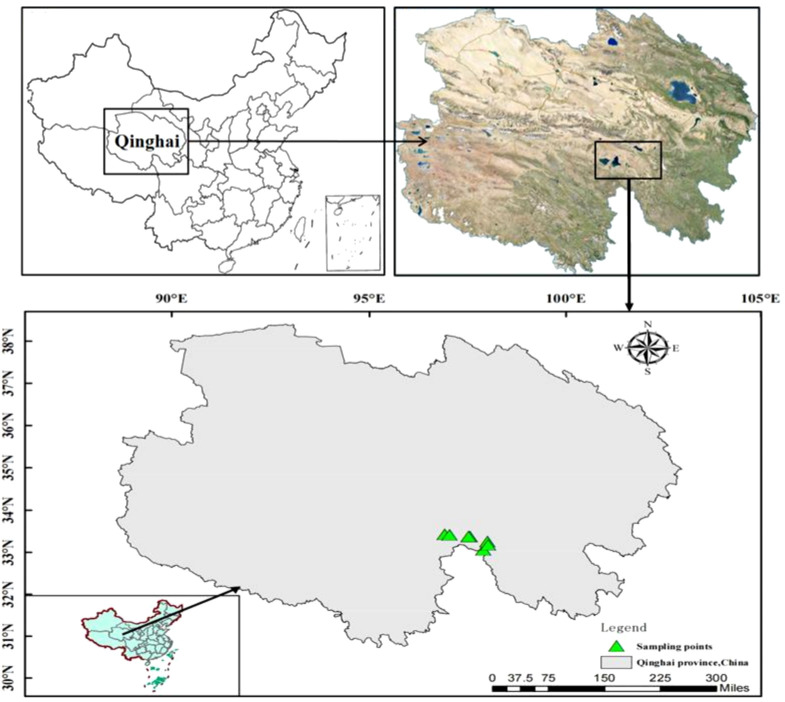
Location map of sampling sites.

At the beginning of September 2021, eight different seismo-faults were selected randomly as sample points based on the locations of the seismic fracture zones and the principle of consistent site conditions including elevation, slope direction, soil parent material, and human management (**Figure 1**). At each sampling site, we used an area approximately 3 m near the seismo-fault as the area affected by the seismic fault zone, and an area not affected by the earthquake on the same slope and same aspect approximately 30 m far from the seismo-fault as the control area. The top 10 dominant species of grassland in control and seismo-fault sites were recorded respectively (**Supplementary Table 1**). In the affected area and control area of each sample site, three areas with a size of 25 cm × 25 cm were randomly selected to collect aboveground plant materials, which were taken back to the laboratory and desiccated at 105°C for 3 h and to a stable weight at 70°C. Then, the dry materials were crushed into fine powder using a vibrating sample mill for subsequent analytical work. Meanwhile, 20-cm-depth soil samples were obtained on the ground at each plant sampling site with soil probes of 3.5 cm diameter. All collected soil samples were then air dried to consistent weight and screened using 0.15-mm sieves.

### 2.3 Laboratory measurement

The contents of total carbon (TC) and total nitrogen (TN) in plants and soil were determined with an elemental analyzer (EA 3000, Italy). The contents of total phosphorus (P), sulfur (S), potassium (K), calcium (Ca), and magnesium (Mg) in plants and soil, as well as the soil available potassium (AK) and available phosphorus (AP) contents, were determined using inductively coupled plasma spectrometry (ICP) (SPECTRO ARCOS EOP, Germany). Soil nitrate (NO_3_^-^-N) and ammonium nitrogen (NH_4_^+^-N) contents were taken by a flow injection auto‐analyzer (AACE, Germany). The total soil organic carbon (SOC) content was measured with a total organic carbon analyzer (TOC-5000A, Japan). The soil pH in the supernatant was measured with a glass electrode, following 5 g of soil homogeneously mixed with 25 mL of water (Shen et al., 2022b).

### 2.4 Statistical analysis

All statistical analyses were carried out using SPSS 26.0. A two-tailed *t*-test was applied to the analysis of the influence on soil properties and plant nutrient content of seismo-faults, and a correlation matrix was developed to determine the relation among soil properties and plant nutrient content. GraphPad Prism 9.3 was used to draw the images. In addition, we developed structural equation modeling (SEM) from the AMOSS 26.0 software package to evaluate the causes of the decline in plant diversity and productivity following the earthquake rupture. We examined the most likely path of influence and corrected the original model with calibration factors, resulting in a qualified SEM. Typically, a competent SEM is required to satisfy the following criteria (Hooper et al., 2007; Jonsson and Wardle, 2010; Wei et al., 2013; Shen et al., 2022a): (1) nonsignificant chi-square test, namely, *p* > 0.05; (2) comparative fit index, CFI > 0.95; and (3) root mean squared error of approximation, RMSEA < 0.05.

## 3 Results

### 3.1 Short-term effects of seismic rupture on soil properties

**Figure 2:**
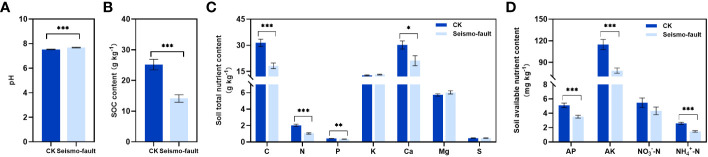
Soil properties change under the influence of seismic rupture. **(A)** Soil pH, **(B)** soil organic carbon content, **(C)** soil total nutrient content, and **(D)** soil available nutrient content. The vertical bars reflect the standard error (SE) of the mean. Significant differences between the control and seismo-fault samples are indicated by asterisks on the SE bars (*p < 0.05, **p < 0.01 and ***p < 0.001).

The negative impacts of earthquakes on soil properties were obvious, with soil properties in the seismo-fault sites differing significantly from those in the control area (**Figure 2**). Seismic rupture caused a significant increase in soil pH (*p* < 0.001) (**Figure 2A**), resulting in soil salinization intensification. Soil total C, N, P, and Ca contents were all greatly reduced by the earthquake rupture (*p* < 0.05), with decreases of 42.00%, 48.63%, 19.27%, and 29.78%, respectively, compared to the control area (**Figure 2C**). The contents of soil available nutrients were also strongly affected by seismic fractures (**Figure 2D**). Soil AP, AK, and NH_4_^+^-N under the seismo-fault were significantly lower than those in the areas not affected by seismic faults (*p* < 0.001), and the decrease rates were 30.91%, 31.58%, and 43.50%, respectively, while the soil NO_3_^-^-N content was not considerably affected by seismic faults (**Figure 2D**). In addition, the seismic rupture had an enormous effect on the SOC content (*p* < 0.001), which decreased by 43.88% in comparison to the control (**Figure 2B**).

### 3.2 Short-term effects of seismic rupture on plant nutrients

**Figure 3:**
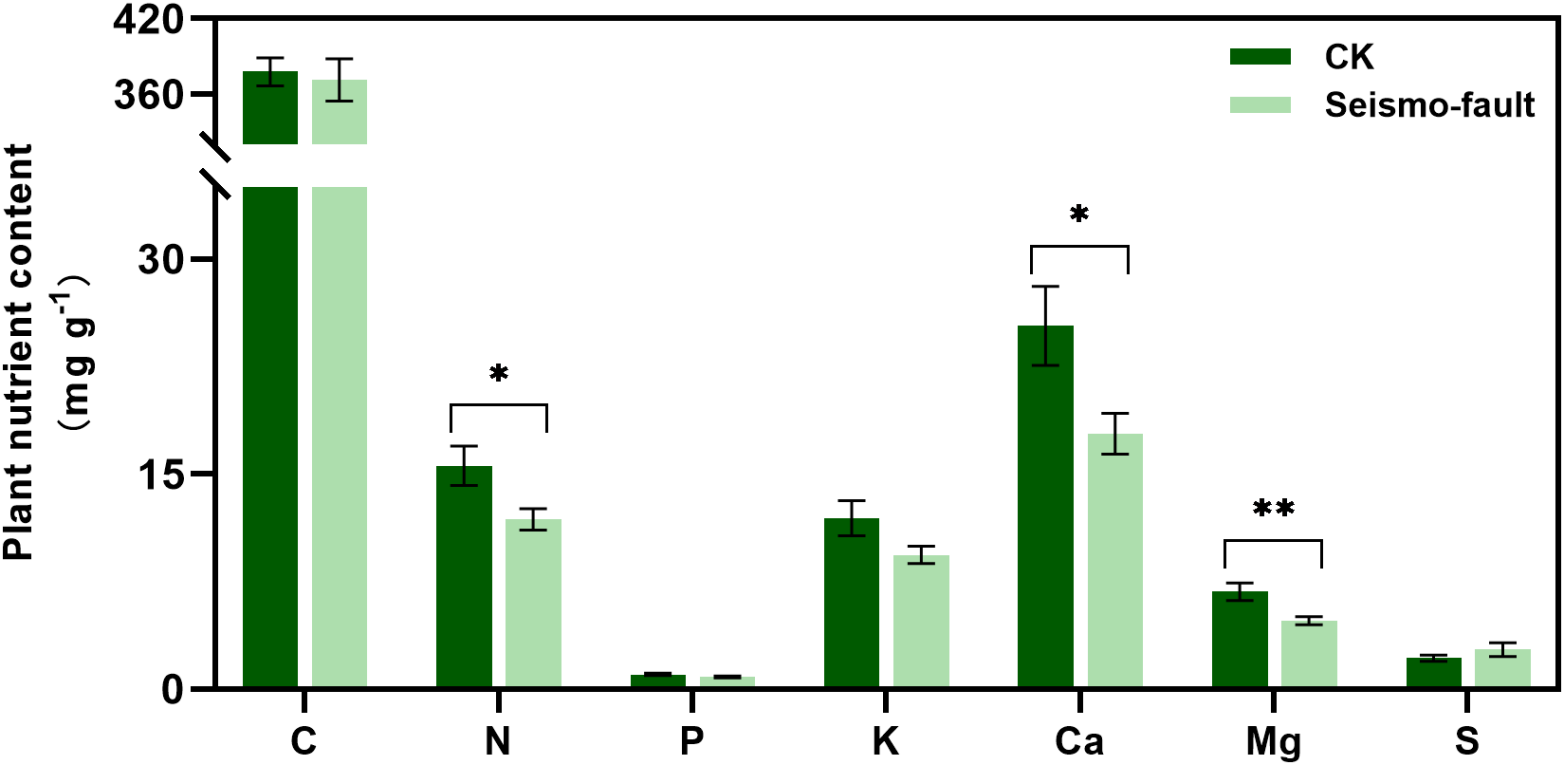
Changes in plant nutrient content under the influence of seismic rupture. The vertical bars reflect the standard error (SE) of the mean. Significant differences between the control and seismo-fault samples are indicated by asterisks on the SE bars (*p < 0.05 and **p < 0.01).

Earthquakes significantly limited plant nutrient uptake. The absorption of N, Ca, and Mg in plants was more sensitive to the short-term effects of the seismic faults. After the earthquake rupture, plant total N, Ca, and Mg contents decreased significantly by 23.97%, 29.73%, and 29.80%, respectively, compared to areas unaffected by the seismo-fault (*p* < 0.05). In contrast, seismic ruptures showed no obvious impact on the total C, P, K, and S contents of plants (**Figure 3**).

### 3.3 Relationship between plant nutrients and soil properties

**Figure 4:**
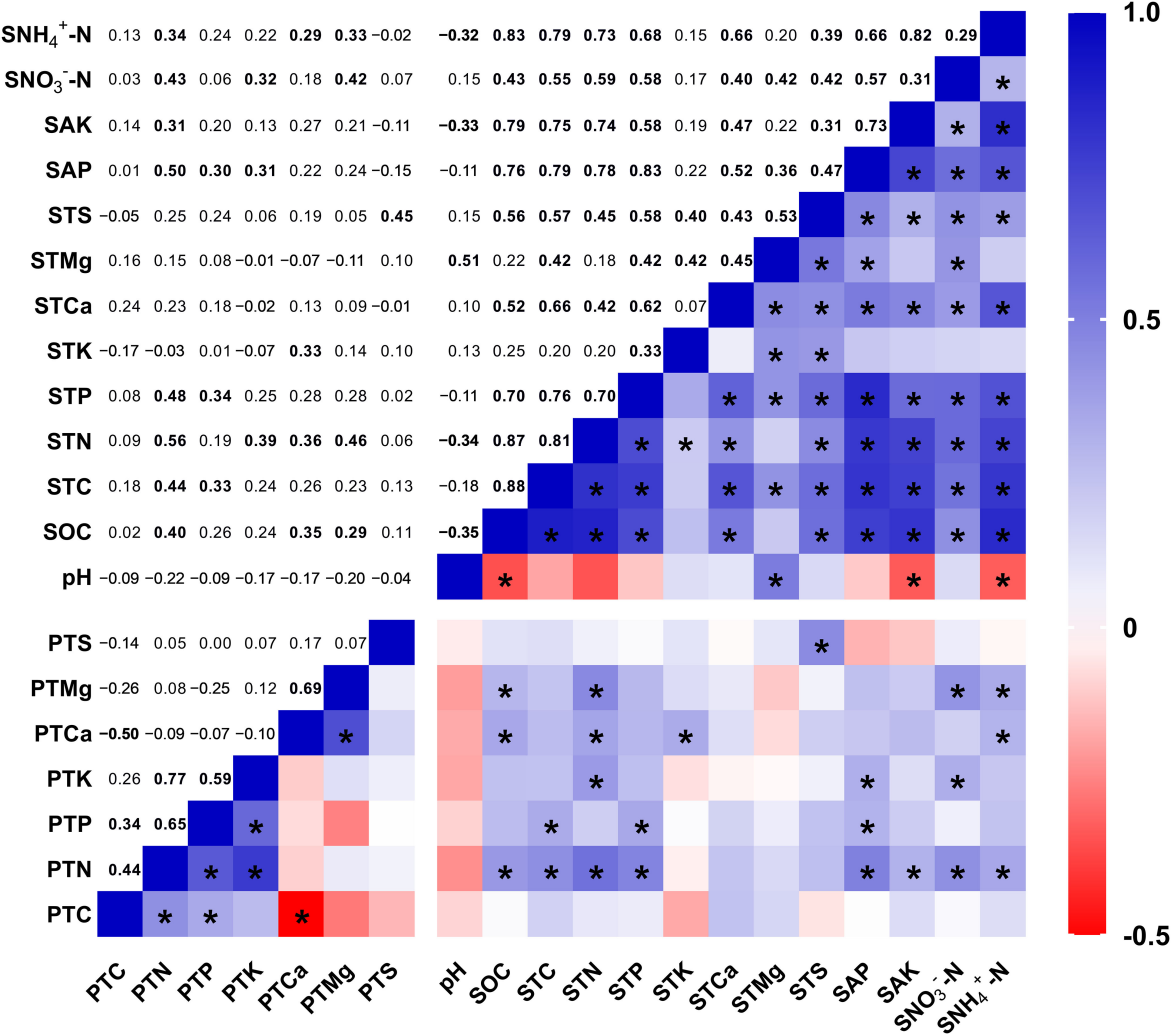
Correlation of plant nutrient content with soil properties. PTC: plant total carbon, PTN, plant total nitrogen; PTP, plant total phosphorus; PTK, plant total potassium; PTCa, plant total calcium; PTMg, plant total magnesium; PTS, plant total sulfur; SOC, total soil organic carbon; STC, soil total carbon; STN, soil total nitrogen; STP, soil total phosphorus; STK, soil total potassium; STCa, soil total calcium; STMg, soil total magnesium; STS, soil total sulfur; SAP, soil available phosphorus; SAK, soil available potassium; SNO_3_^–^N, soil nitrate nitrogen; SNH_4_^+^-N, soil ammonium nitrogen. * and bolded numbers mean significant differences at the level of *p* < 0.05.

Using data from seismic fault zones and control areas, we evaluated the link between plant nutrient indicators and soil properties using a Pearson correlation matrix (**Figure 4**). The findings revealed that the plant nutrient contents, which had been significantly altered by the short-term effects of the earthquake rupture, were closely related to the soil nutrient contents (**Figure 4**). Plant N content was shown to be strongly and positively associated to SOC (*r* = 0.40, *p* < 0.01), soil TC (*r* = 0.44, *p* < 0.01), soil TN (*r* = 0.56, *p* < 0.001), soil TP (*r* = 0.48, *p* < 0.001), soil AP (*r* = 0.50, *p* < 0.001), soil AK (*r* = 0.31, *p* < 0.05), soil NO_3_^-^-N (*r* = 0.43, *p* < 0.01), and NH_4_^+^-N (*r* = 0.34, *p* < 0.05) contents. Plant Ca content had a great positive connection with SOC (*r* = 0.35, *p* < 0.05), soil TN (*r* = 0.36, *p* < 0.05), soil TK (*r* = 0.33, *p* < 0.05), and soil NH_4_^+^-N (*r* = 0.29, *p* < 0.05) contents. Plant Mg content demonstrated a significant positive association with regard to SOC (*r* = 0.29, *p* < 0.05), soil TN (*r* = 0.46, *p* < 0.001), soil NO_3_^-^-N (*r* = 0.42, *p* < 0.01), and NH_4_^+^-N (*r* = 0.33, *p* < 0.05) contents. In addition, the plant Ca and Mg content displayed a strong positive association (*r* = 0.69, *p* < 0.001). Although the pH of soil had no direct correlation with plant nutrient contents, it showed a significant inverse relationship with SOC (*r* = −0.35, *p* < 0.05), soil AK (*r* = −0.33, *p* < 0.05), and soil NH_4_^+^-N (*r* = −0.32, *p* < 0.05) contents and a significant positive correlation with total soil Mg (*r* = 0.51, *p* < 0.001), thus possibly indirectly influencing plant nutrient contents.

### 3.4 Direct and indirect drivers associated with earthquake affecting plant productivity and diversity

**Figure 5:**
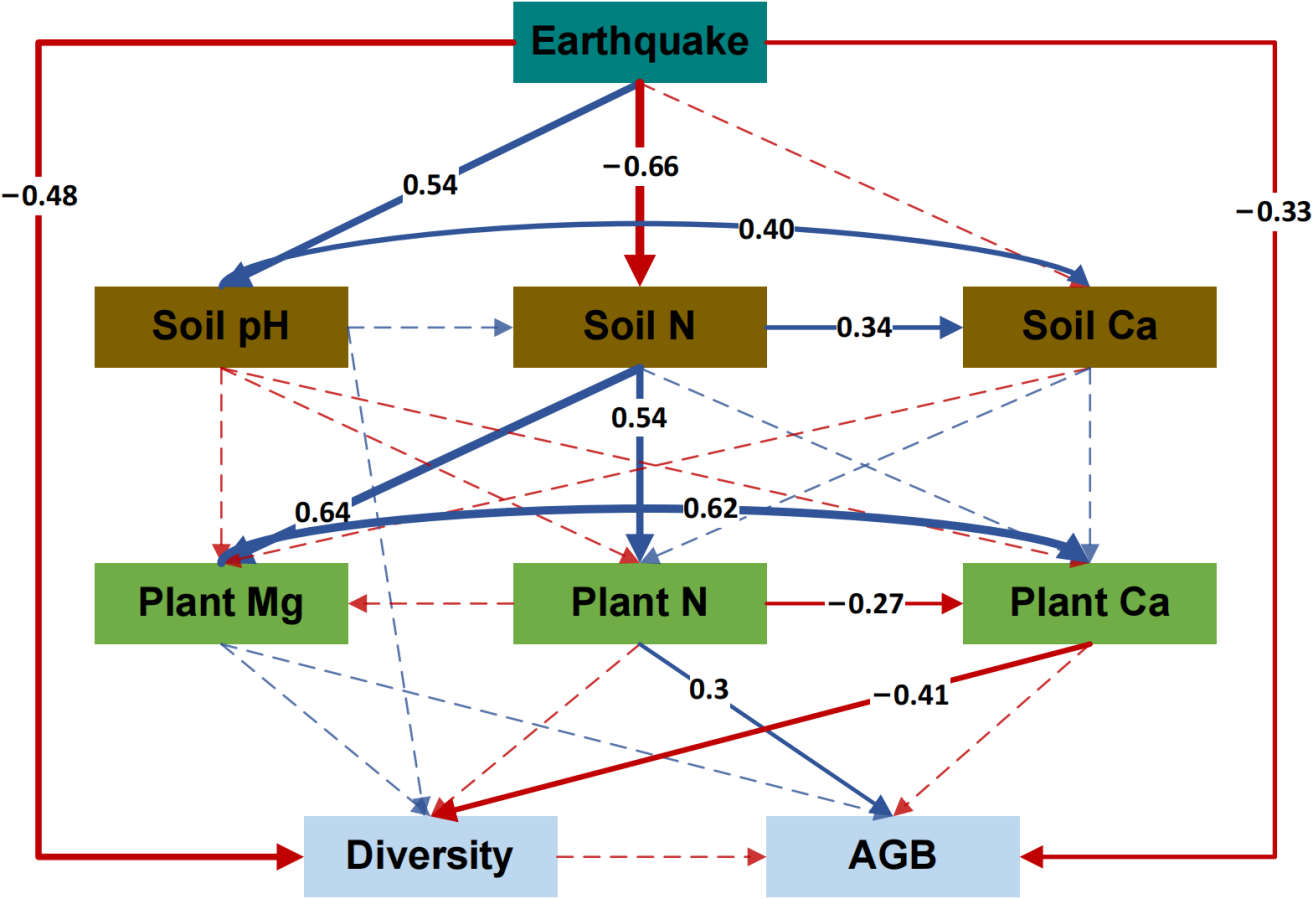
Structural equation model (SEM) analysis of the influence path of earthquake on plant community diversity and productivity. Significant positive relationships are denoted by the solid blue lines (*p* < 0.05), while significant negative relationships are indicated by the solid red lines (*p* < 0.05). Weak positive or negative relationships are indicated by dashed blue or red lines, respectively (*p* > 0.05). Arrow width varies directly with the path coefficient’s strength. Soil N, soil total nitrogen content; Soil Ca, soil total calcium content; Plant N, plant total nitrogen content; Plant Mg, plant total magnesium content; Plant Ca, plant total calcium content; Diversity, Shannon–Wiener index of the plant community; AGB, aboveground biomass of the plant community. Chi/df = 1.025, *p* = 0.414, GFI = 0.963, CFI = 0.998, RMSEA = 0.023.

Earthquakes significantly affected the diversity and productivity of alpine grassland communities. In **Supplementary Figure 1**, it is evident that seismo-fault obviously reduced the Shannon–Wiener Index and aboveground plant biomass of alpine grassland communities compared to the control area, indicating that the earthquake led to a decline in grassland plant community diversity and a reduction in productivity. Therefore, an SEM was developed to explore the impacts of seismic rupture on plant community diversity and productivity (**Figure 5**). The results showed that seismic ruptures affected plant community diversity and productivity through different pathways. First, earthquake rupture had a clear direct impact on both the diversity and productivity decline of plants. Although the reduction in soil nutrient content caused by seismo-faults inhibited the absorption of Ca by plants, and the plant Ca content showed a negative relationship with community diversity, the direct effect of seismo-faults was stronger and significantly reduced community diversity. In addition, seismo-faults also affected community productivity through more complex indirect pathways. Seismic fractures inhibited plant uptake of N mainly by affecting the soil N content, thus decreasing the plant N content and reducing aboveground biomass.

## 4 Discussion

### 4.1 Earthquake limited plant nutrient uptake by affecting the soil nutrient balance

As a reservoir of nutrients required by plants, soil nutrient changes have an important role to play for plant growth and development (Roca-Pérez et al., 2002; Chaudhary et al., 2008; Friedel and Ardakani, 2020). Undisturbed and well-structured soils, the basic foundation of most of the world’s plants, maintain the sustainability, stability, and balance of ecosystems (Rodríguez Rodríguez et al., 2005). Nevertheless, the original nutrient-rich and well-formed soils are susceptible to earthquake disturbance, which destroys the topsoil structure and causes serious soil erosion, further leading to soil nutrient loss (Vittoz et al., 2001; Cui et al., 2012). Our results also suggested that earthquakes greatly contributed to soil nutrients loss, which is in agreement with our first hypothesis. The contents of soil TC, TN, TP, TCa, AP, AK, NH_4_^+^-N, and SOC were significantly decreased compared with those in areas not affected by the earthquake. This was in keeping with findings by Guo et al. (2013) and Lin et al. (2017) that soil total nutrient content was greatly lost following earthquake, and the contents of SOM (soil organic matter), TN, TP, AHN (alkaline hydrolyzable nitrogen), AP, and AK in the soil were significantly lower in comparison to areas not disturbed by the earthquake. Qiu et al. (2022) also found that soil TC, TP, TN, TS, AP, NO_3_^-^-N, NH_4_^+^-N, and SOC contents were reduced under alpine wetland environmental conditions after the Maduo earthquake. These nutrient losses may be the result of soil loosening brought on by earthquakes and other secondary geological hazards, which leads to increased aerobic microbial growth and activity, thus speeding up the decomposition of organic matter (Walker and Shiels, 2008; Pupin et al., 2009). Soil pH, an important indicator characterizing soil acidity and alkalinity, strongly influences plant activity (Tsai and Schmidt, 2021). Our findings indicated that earthquakes caused an increase in soil alkalinity and a rise in pH. This phenomenon was also confirmed by the study of Lin et al. (2017). This may be caused by the change in soil profile structure after the earthquake, with deeper layers of carbonate exposed at the surface, leading to an increase in soil pH. An increase in soil pH will raise the negative charge on the surface of plant roots, thus affecting the utilization for nutrients by the plant (Nautiyal et al., 2000; Chaudhary et al., 2008; Barrow and Hartemink, 2023).

Soil nutrients are closely related to plant nutrients. Soil nutrient content is critical to plant growth and is considered a key variable and external factor in plant response (Achat et al., 2018; Briat et al., 2020). The plant root system can take up nutrients from the soil directly, or indirectly from the soil and atmosphere through symbiosis with inter-rooted microorganisms (Patriarca et al., 2002; Katayama et al., 2014). The change in the nutrient content of the soil is the major factor controlling nutrients in plant (Güsewell and Koerselman, 2002; Wang et al., 2018; Zhang et al., 2019). Following earthquake, total and available nutrient contents in the soil were significantly reduced, which led to a decrease in the nutrients available to plants and ultimately led to a loss of nutrient content in the plants. Of these, plants were more sensitive to N, Ca, and Mg uptake, with significant decreases. In arid ecosystems, soil–vegetation relationships were more associated with Ca, N, and Mg, which confirmed our findings (Pena-Claros et al., 2012). SEM analysis also showed that soil total N played a major role in affecting plant nutrient uptake, which was in agreement with the study of Zhang et al. (2019), where the main factor affecting plant nutrient content was soil total N (Zhang et al., 2019). In short, the earthquake limited the absorption of plant nutrients by affecting soil nutrients, which consequently prevented growth of plants and the development and stability of grassland communities, resulting in profound negative effects on grassland ecosystems. This confirmed our second hypothesis.

### 4.2 Earthquake directly affected grassland plant diversity

The diversity of plant species is determined by a range of factors at various scales of space (Pena-Claros et al., 2012). Among environmental conditions, soil type and topography have a key part in forming plant diversity, as they both affect the effectiveness of water and nutrients (Potts et al., 2002; Phillips et al., 2003). The structure and diversity of the plant community are influenced by soil nutrients and soil texture in different ways (Pena-Claros et al., 2012). In our research, the decrease in soil nutrients after the earthquake led to a decrease in TN, TCa, and TMg in plants. The nutritional status of plants within a community is closely related to species richness. In some cases, an increase in the nutrient content available to plants will lead to a corresponding decline in species richness. Because of the more limited plant resources, the more niche separation, the greater opportunities for specific trade-offs between different species, the greater the possibility of avoiding competition, and ultimately the coexistence of more plant species (Harpole and Tilman, 2007; Klaus et al., 2013; Borer et al., 2014). Studies have demonstrated that in grassland ecosystems, N and P contents in plant leaves were negatively linked to species richness (Lü et al., 2019) and had a direct impact on plant richness (Harpole and Tilman, 2007). This was similar to our findings, which showed that the N content in plants was negative but not significantly with plant community diversity, while the Ca content was strongly negative with plant community diversity. This may be because plants tend to maintain high community diversity by maintaining low nutrient concentrations in species and improving nutrient utilization efficiency (van Ruijven and Berendse, 2005; Selmants et al., 2014; Lü et al., 2019). However, the low nutrient content of plants in our study did not sustain the increase in diversity, which may be because the decrease in nutrient levels in plants was due to the restriction of lower nutrient content in the soil, rather than the selective absorption strategies of the plants themselves. Grassland community composition is closely related to the concentration of Ca in aboveground biomass, as numerous legumes are accumulators of Ca (Jakobsen, 1993; Bauer et al., 2011). Legumes require higher levels of Ca for the best development (Broadley et al., 2004). This also agrees with our earlier study, i.e., the importance values of legumes decreased significantly after the earthquake rupture (Zuo et al., 2022). Ca levels in plants are highly changeable, but monocots typically have lower Ca concentrations than eudicots, suggesting that low Ca concentrations are more suitable for the growth of monocots like grasses (Broadley et al., 2003). However, the biomass of graminoids was strongly and favorably associated with N content, and the earthquake decreased soil N content, so grassland plant diversity was not enhanced by the decrease in calcium content in plants. Conversely, although a decline in Ca may lead to a reduction in some calciphilous plants and to some extent favor the growth of calciphobe plants, the direct negative short-term effects of earthquakes have a negative impact on all plants and ultimately lead to a decline in diversity. This suggests that the direct effect of the earthquake on decreasing species diversity is significant. Contrary to our third hypothesis, the decrease in plant community diversity from earthquakes may be mainly caused by direct effects such as changes in soil spatial structure.

Several earthquake fault zones were formed directly in the alpine grasslands of the QTP following the M*w* 7.4 Maduo earthquake in 2021. This disruption to the soil structure caused severe soil erosion, leading to an increase in soil permeability and infiltration capacity and a reduction in hydrological regulation, drainage, capacity, and temperature (Vittoz et al., 2001; Cui et al., 2012). In terms of physical properties, the earthquake changed the composition of soil particles, leading to higher sand and clay contents and a decrease in soil moisture content, which replaced previously well-formed soils (Lin et al., 2017), further leading to a decrease in plant community diversity. It has been shown that the Maduo earthquake led to seepage of surface water close to seismic fissures, which caused a reduction in surface water or complete drying, resulting in a reduction in soil moisture, an increase in soil hardness and salinity, and ultimately a loss of vegetation (Qiu et al., 2022).

### 4.3 Plant nutrient limitation following an earthquake contributed greatly to community productivity reduction

The interaction between plants and soil nutrients shapes plant community structure, which also has a further impact for the productivity of plants (Silva and Batalha, 2008; Wang et al., 2009). Many works have demonstrated that the accessibility of plant nutrients is one of the key elements influencing plant productivity (Elser et al., 2007; Harpole and Tilman, 2007; Fay et al., 2015). Klaus et al. (2013) found that nutrient contents in plants were strongly and positively linked to aboveground biomass and that nutrients were often synergistically co-limited to productivity (Fay et al., 2015). Of these, N is considered a critical determination of aboveground net primary production (ANPP) (Vitousek and Howarth, 1991; LeBauer and Treseder, 2008). This is because N content in plants, an essential nutrient for photosynthetic organ building, is strongly correlated with root tip length or leaf area, while the leaf area index in turn is significantly correlated with total primary productivity (Koller et al., 2015). In agreement with these findings, our study revealed that strong earthquakes caused a decrease in productivity not only through direct effects such as damage to soil structure but also through a decrease in soil N content, limiting plant N uptake and further affecting plant community and productivity. This also confirmed our third hypothesis. Moreover, we can further confirm that N is not only the principal nutrient limiting plant development in grassland ecosystems but also a limiting factor for primary productivity in grassland ecosystems and an essential indicator of grassland productivity (Vitousek et al., 1997; Koller et al., 2015).

## 5 Conclusion

Our findings showed that the strong earthquake significantly reduced soil quality in the short term, caused soil nutrient loss, and inhibited plant nutrient uptake. The direct negative effect of earthquake was the major cause of the decline in grassland plant diversity in alpine grassland ecosystem on the QTP. Furthermore, in addition to the direct impact pathways of earthquake, earthquake can reduce the productivity of grassland community through the indirect pathway of loss of soil nutrients and inhibition of plant nutrient uptake. The decrease in soil N content after the earthquake significantly inhibited plant nutrient uptake, leading to a significant decrease in plant community productivity. In summary, the earthquake damaged the functional stability of the alpine grassland ecosystem on the QTP. Effective management and restoration methods are desperately required to strengthen the stability and health of grassland ecosystems following strong earthquakes.

## Data availability statement

The raw data supporting the conclusions of this article will be made available by the authors, without undue reservation.

## Author contributions

HZ: Formal analysis and Writing—original draft. HShen: Writing—review and editing. SD: Writing—review and editing. SW: Investigation. FH: Investigation. RZ: Investigation. ZW: Investigation. HShi: Investigation. XH: Investigation. YT: Investigation. CM: Investigation. SL: Investigation. YL: Investigation. FZ: Investigation. JX: Investigation. All authors contributed to the article and approved the submitted version.
