# Supplementary material for: Potential short-term effects of earthquake on the plant–soil interface in alpine grassland of the Qinghai–Tibetan Plateau
Source: Front Plant Sci. 2023 Oct 17;14:1240719. doi: 10.3389/fpls.2023.1240719 (PMC10616788; doi:10.3389/fpls.2023.1240719)
Supplement: Supplementary file 1 [file DataSheet_1.docx]

**Supplementary Material**

## Supplementary Figures

**
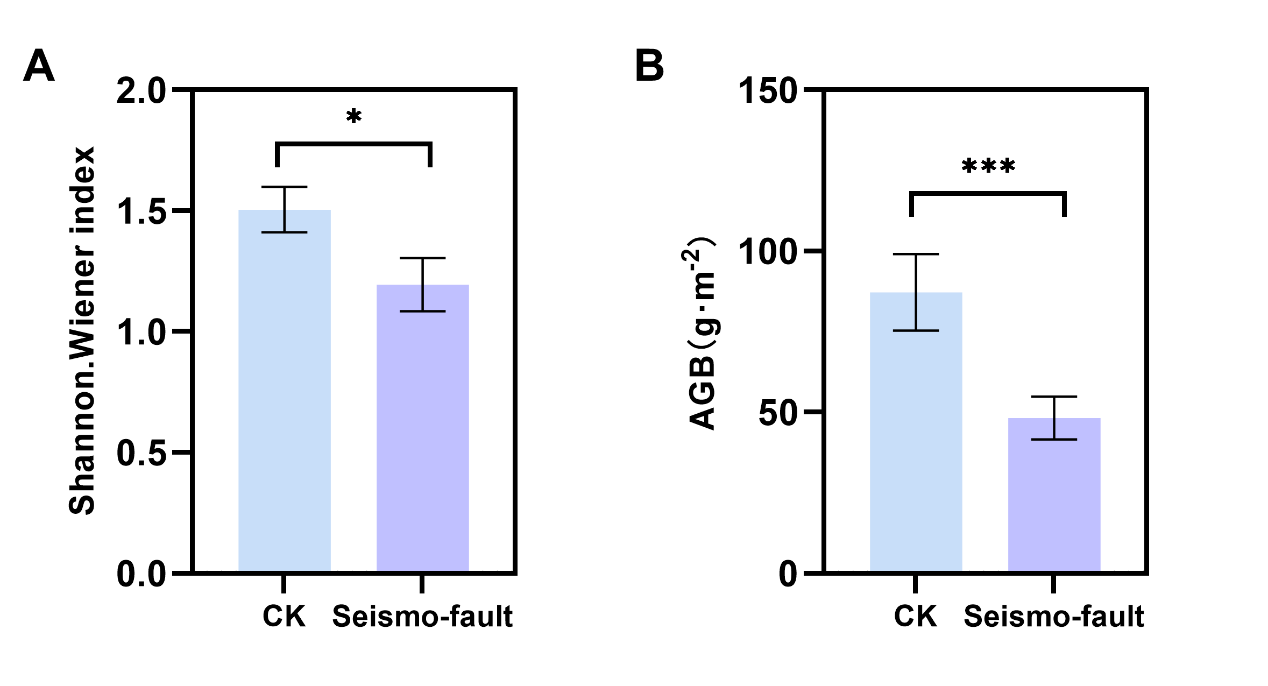
**

**FIGURE S1|** The effect of seismic rupture on diversity index and aboveground productivity. (A) Shannon-Wiener index and (B) aboveground plant biomass. Vertical bars represent the standard error (SE) of the mean. Asterisks on the SE bars indicate significant differences between the control and seismo-fault samples (**P*<0.05 and *** *P*<0.001).

## Supplementary Tables

**Table S1|** Dominant species under the influence of seismic rupture.

| **CK** | | | | |  | **Seismo-fault** | | | |
| --- | --- | --- | --- | --- | --- | --- | --- | --- | --- |
|  | **Family** | **Species** | **Life form** | **Functional group** |  | **Family** | **Species** | **Life form** | **Functional group** |
| 1 | Cyperaceae | *Kobresia humilis* | Perennial | Sedges | 1 | Polygonaceae | *Polygonum sibiricum* | Perennial | Forbs |
| 2 | Cyperaceae | *Kobresia pygmaea* | Perennial | Sedges | 2 | Primulaceae | *Glaux maritima* | Perennial | Forbs |
| 3 | Cyperaceae | *Carex scabrirostris* | Perennial | Sedges | 3 | Compositae | *Artemisia capillaris* | Perennial | Forbs |
| 4 | Compositae | *Artemisia capillaris* | Perennial | Forbs | 4 | Cyperaceae | *Carex scabrirostris* | Perennial | Sedges |
| 5 | Gramineae | *Poa pratensis* | Perennial | Grasses | 5 | Gramineae | *Leymus secalinus* | Perennial | Grasses |
| 6 | Suoicacae | *Salix cupularis* | perennial | Others | 6 | Cyperaceae | *Kobresia pygmaea* | Perennial | Sedges |
| 7 | Primulaceae | *Glaux maritima* | Perennial | Forbs | 7 | Scrophulariaceae | *Lancea tibetica* | Perennial | Forbs |
| 8 | Gramineae | *Leymus secalinus* | Perennial | Grasses | 8 | Compositae | *Artemisia frigida* | Perennial | Forbs |
| 9 | Compositae | *Leontopodium leontopodioides* | Perennial | Forbs | 9 | Gentianaceae | *Gentiana straminea* | Perennial | Forbs |
| 10 | Gramineae | *Agropyron cristatum* | Perennial | Grasses | 10 | Liliaceae | *Allium przewalskianum* | Perennial | Forbs |
